# Evaluation of ECHO PS Positioning System in a Porcine Model of Simulated Laparoscopic Ventral Hernia Repair

**DOI:** 10.1155/2013/862549

**Published:** 2013-05-23

**Authors:** Erin M. Hanna, Guy R. Voeller, J. Scott Roth, Jeffrey R. Scott, Darcy H. Gagne, David A. Iannitti

**Affiliations:** ^1^Department of Surgery, Carolinas Medical Center, Charlotte, NC 28270, USA; ^2^Department of Surgery, University of Tennessee Health Science Center, Memphis, TN 38163, USA; ^3^Department of Surgery, University of Kentucky College of Medicine, Lexington, KY 40536, USA; ^4^Department of Molecular Pharmacology, Physiology & Biotechnology, Brown University, Providence, RI 02906, USA; ^5^C. R. Bard, Inc. (Davol), Warwick, RI 02886, USA

## Abstract

*Purpose*. Operative efficiency improvements for laparoscopic ventral hernia repair (LVHR) have focused on reducing operative time while maintaining overall repair efficacy. Our objective was to evaluate procedure time and positioning accuracy of an inflatable mesh positioning device (Echo PS Positioning System), as compared to a standard transfascial suture technique, using a porcine model of simulated LVHR. *Methods*. The study population consisted of seventeen general surgeons (*n* = 17) that performed simulated LVHR on seventeen (*n* = 17) female Yorkshire pigs using two implantation techniques: (1) Ventralight ST Mesh + Echo PS Positioning System (Echo PS) and (2) Ventralight ST Mesh + transfascial sutures (TSs). Procedure time and mesh centering accuracy overtop of a simulated surgical defect were evaluated. *Results*. Echo PS demonstrated a 38.9% reduction in the overall procedure time, as compared to TS. During mesh preparation and positioning, Echo PS demonstrated a 60.5% reduction in procedure time (*P* < 0.0001). Although a trend toward improved centering accuracy was observed for Echo PS (16.2%), this was not significantly different than TS. *Conclusions*. Echo PS demonstrated a significant reduction in overall simulated LVHR procedure time, particularly during mesh preparation/positioning. These operative time savings may translate into reduced operating room costs and improved surgeon/operating room efficiency.

## 1. Introduction

 Laparoscopic ventral hernia repair (LVHR) has gained acceptance as a safe and effective alternative to open ventral hernia repair (OVHR), resulting in reduced patient complications and hospital stays [1, 2]. Previous reports have demonstrated procedure time for LVHR to be equivalent or less than OVHR, and there has been an increasing trend towards improvement of LVHR operative efficiency by reducing procedure time/cost while maximizing the aforementioned patient benefits and overall hospital efficiency [1, 3, 4]. A focus on cost effective time management strategies in the operating room has come to the forefront as one area for improvement in healthcare expenditures [5]. 

One approach for reduction of operative procedure costs associated with LVHR would be to reduce overall procedure time by improving procedure efficiency. In an example of improved procedure efficiency, a team-based approach was implemented by John's Hopkins University to improve overall efficiency in percutaneous tracheostomy; the resulting expenditure analysis was found to produce significant financial benefits from savings realized on multiple levels [6]. A second approach employs device-based methods for improvement in operative efficiency; this type of cutting edge technology bears the significant task of demonstrating considerable improvement in operative efficiency in order to gain widespread, mainstream acceptance. Previously introduced devices for efficiency improvement in laparoscopic surgery have focused on particularly challenging procedural aspects such as intracorporeal suturing. 

**Figure 1:**
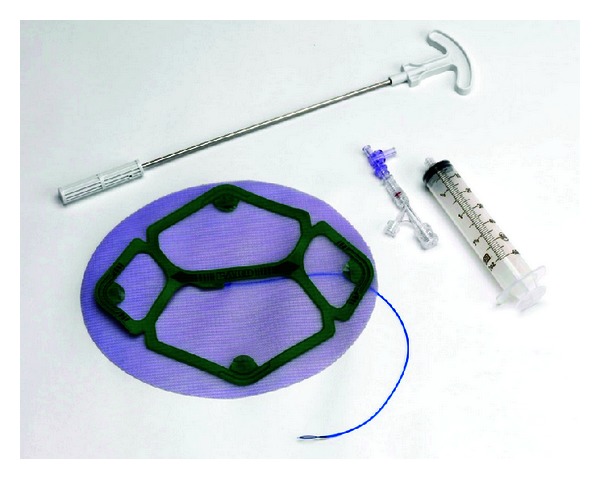
Ventralight ST Mesh with Echo PS Positioning System.

Recently introduced to the market, a new product designed to stabilized and support mesh during LVHR also has the potential for efficiency improvement and time savings in the operating room. Echo PS Positioning System (Echo PS) (C. R. Bard, Inc. (Davol), Warwick, RI, USA) employs a user inflated balloon temporarily affixed to the mesh to hold the mesh taut and flat while conforming the to abdominal wall. By eliminating many of the steps needed for transfascial suture placement and positioning, Echo PS has the potential to decrease operative time during one of the most challenging portions of the operation, orientation, unrolling, and initial fixation of the mesh. In this study, we utilize a porcine animal model of simulated LVHR and asked experienced general surgeons to evaluate overall differences in time to perform simulated LVHR using a standard transfascial suture technique, as compared to LVHR using the new Echo PS device (Figure 1). 

## 2. Methods

 To appropriately simulate the operative environment and *in vivo* LVHR, we elected to perform this study using a large porcine animal model. Approval for this study was obtained from the Institutional Animal Care and Use Committee at CBSET, Inc. (Lexington, MA, USA). All animals were treated in accordance with the *Guide for the Care and Use of Research Animals*. Female Yorkshire pigs (80–90 kg) were housed in standard pens and given standard water and chow until the day prior to surgery. Surgeons were consented to participate in an *in vivo* preclinical simulation project, which included the capturing of procedural metrics, photography, video recordings, and questionnaire data in a nonidentifiable format.

**Figure 2:**
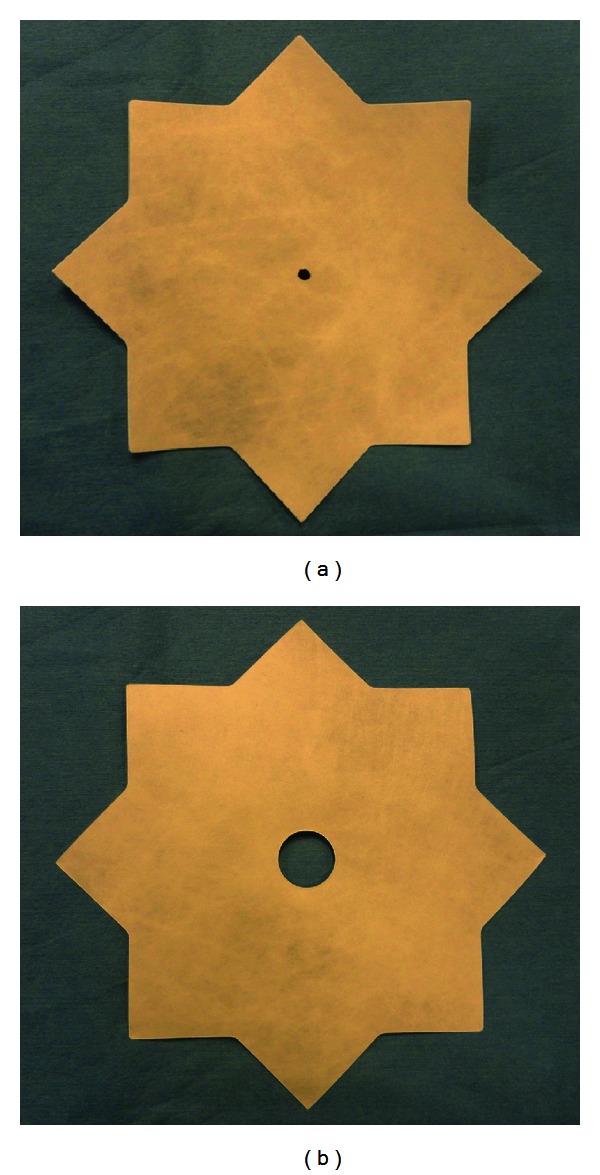
(a) Intraabdominal Tyvek template used to create a 10′′ circle simulated hernia defect (fasteners placed at the vertices); (b) external Tyvek template used to transfer corresponding surgical defect markings onto the external abdominal wall.

**Figure 3:**
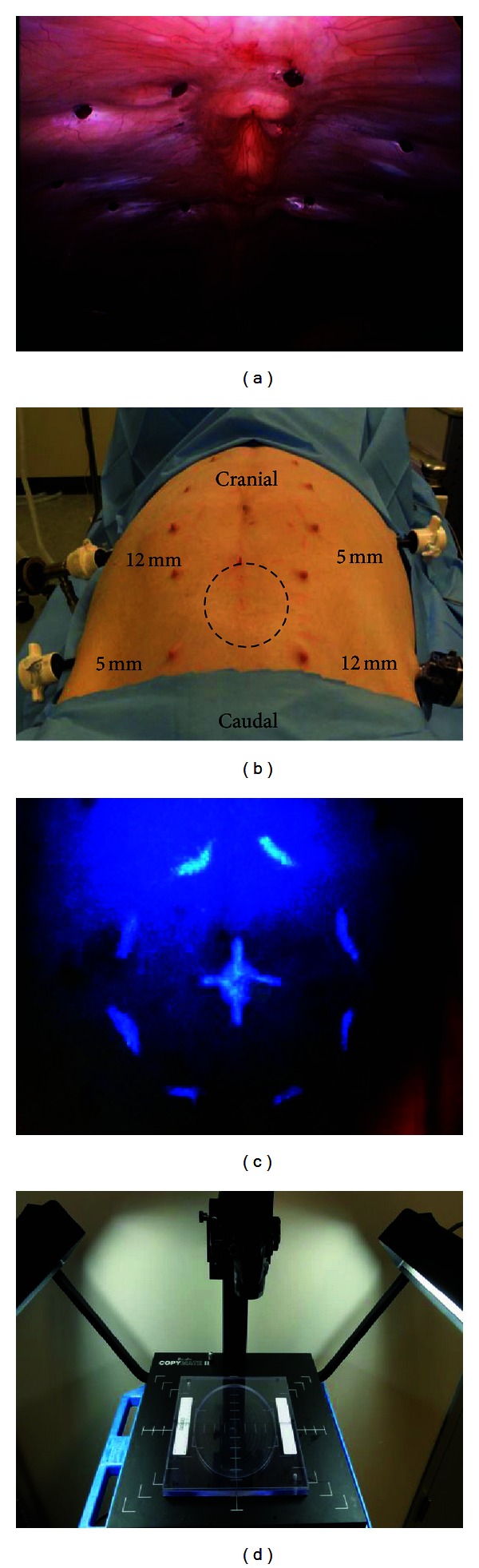
(a) Simulated 10′′ circle surgical defect marked on the parietal surface of the abdominal wall with Sorba FIX fasteners, (b) four trocars were placed in the abdominal wall (two 5 mm and two 12 mm), (c) the center of the simulated surgical defect was marked on the external abdominal wall with black light sensitive ink, and (d) photography station was used for assessing the accuracy of mesh placement after removal from the abdomen.

Animal anesthesia was induced by injection of Telazol (4–6 mg/kg IM) as a preanesthetic. Isoflurane anesthesia was delivered in 100% oxygen to facilitate endotracheal intubation. Animals were maintained under maintenance anesthesia with inhaled isoflurane and were then placed in dorsal recumbency, and the abdomen was insufflated using a Veress needle. Four trocars were placed in the abdominal wall: two 5 mm trocars and two 12 mm trocars in lateral subxiphoid positions (Figure 3(b)). A simulated ventral hernia defect measuring 10 cm in diameter was placed using a template (Figure 2(a)) on the medial parietal peritoneum surface of the porcine abdominal wall (Figure 3(a)) and marked using laparoscopically deployed Sorba FIX fasteners (C. R. Bard, Inc. (Davol), Warwick, RI, USA). To denote the location of the center of the simulated surgical defect, a second template was overlaid on the outer abdominal wall and the centers of each target aligned (Figure 2(b)). The simulated defect center was then marked on the outer abdominal wall using a black light sensitive marker (non-identifiable to the surgeons) for identification following LVHR simulation (Figure 3(c)). Mesh size was standardized for all simulated LVHR at 8 × 10 in (20.3 × 25.4 cm) which given the 10 cm simulated surgical defect would allow for a minimum target of 5 cm of overlap in each direction.

**Figure 4:**
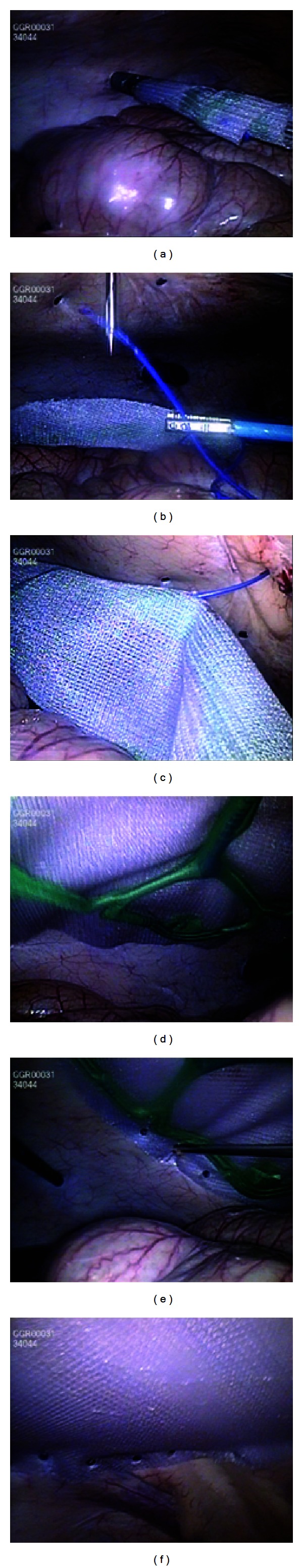
Placement of Ventralight ST Mesh with Echo PS. (a) Mesh trocar insertion with the introducer tool, (b) capture of the central inflation tube with a suture passer, (c) inflation tube passed through the center of the simulated surgical defect, (d) Echo PS device inflation, (e) initial fixation with Sorba FIX fasteners, and (f) Echo PS device removal through a 5 mm trocar site.

**Figure 5:**
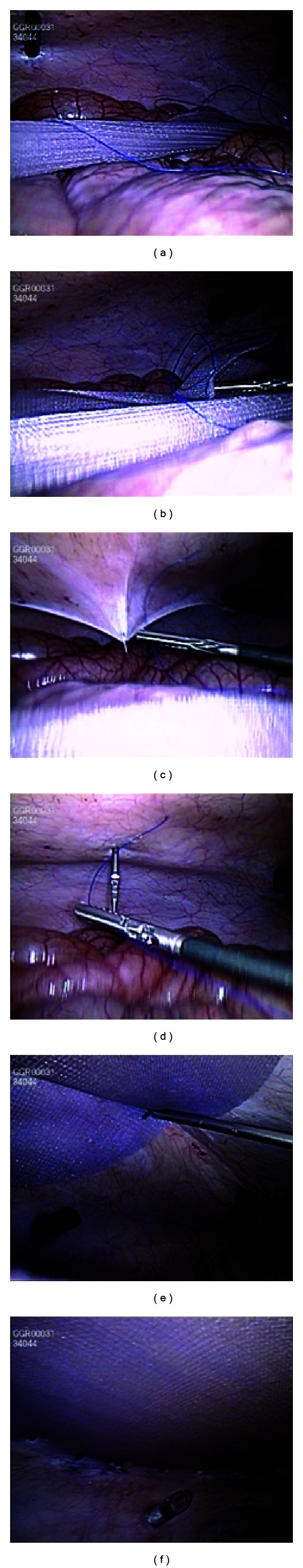
Placement of Ventralight ST Mesh with transfascial sutures. (a) Preplacement of four sutures and mesh trocar insertion with a laparoscopic grasper, (b) mesh unrolling, flattening, and orientation, (c, d) identification of preplaced sutures and passage of each suture through the abdominal wall for positioning, and (e, f) fixation with Sorba FIX fasteners.

Each surgeon served as their own control and performed simulated LVHR using Ventralight ST Mesh with Echo PS Positioning System (Echo PS) (C. R. Bard, Inc. (Davol), Warwick, RI, USA) (Figure 4) and Ventralight ST Mesh (C. R. Bard, Inc. (Davol), Warwick, RI, USA) with transfascial sutures (Figure 5). Surgeons were randomized as to which LVHR technique was performed first. The same surgical scrub and assistant were utilized for each surgeon. Surgeons were blinded as to the study end points. Instructions were provided to identify the hernia defect and measure/mark approximate locations of transfascial sutures on the abdominal wall or in the case of Echo PS, the location for the center positioning tubing. Surgeons were then instructed to insert, position, and fixate mesh using a Sorba Fix laparoscopic fixation device. The number of fasteners used to secure each mesh was standardized at 24 (placed circumferentially) per mesh. Ventralight ST Mesh with transfascial sutures was prepared by the test surgeon with four prolene sutures, which were preplaced in each of the four cardinal directions. Transfascial sutures were used to position the mesh and were pulled through the abdominal wall using a Storz suture passer and sutures were left clamped at the level of the skin with hemostats. Following completion of the two LVHR simulations, surgeons were given an opinion-based questionnaire to assess previous training, familiarity with the Echo PS device, frequency of similar procedures performed, and ease of use in the testing scenario.

**Figure 6:**
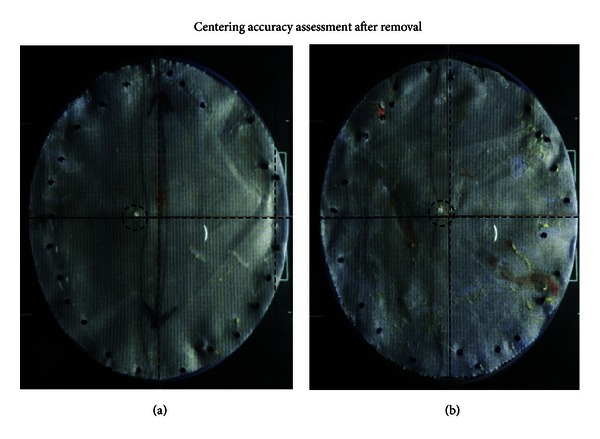
Mesh centering accuracy assessment after removal. (a) Ventralight ST Mesh with transfascial sutures; (b) Ventralight ST Mesh with Echo PS. Circled areas represent a white Perma Fix fastener that was laparoscopically placed at the center of simulated surgical defect. The distance between the center of the mesh and the center of the simulated surgical defect was defined as “center error”.

 Time points were recorded from when the surgeon first touched the mesh until the final fastener was deployed to secure the mesh to the abdominal wall. Data points collected included total procedure time from initial mesh preparation through the completion of mesh fixation or the removal of the Echo PS balloon. In addition, mesh positioning time was also collected. This was recorded from the time the mesh entered the abdomen to the placement of the first fastener. Following mesh fixation using either technique, a black light was used to identify the center point of the simulated surgical defect, a spinal needle passed through that point, and the center was marked on the mesh laparoscopically using a white Perma Fix fastener (C. R. Bard, Inc. (Davol), Warwick, RI, USA). The mesh was then removed from the abdominal wall and placed into a photography station for centering accuracy measurement (Figure 3(d)). A calibrated caliper was then used to measure the center point marked with the Perma Fix fastener to the true center of the mesh to evaluate the center error (Figure 6). Upon completion of the first procedure, the same surgeon would complete the second procedure, and all of the aforementioned metrics would be captured in an identical fashion. Upon completion of the second mesh fixation, the animal was euthanized utilizing accepted veterinary methods.

 Statistical analysis was performed using GraphPad Prism (GraphPad Software, La Jolla, CA, USA) and SAS version 9.2 (SAS Institute, Cary, NC, USA). Descriptive statistics, including means and standard errors/deviations or counts and percentages, were calculated. Data were measured on an interval scale, and the paired Student's *t*-test was used to compare the two groups. The Wilcoxon rank-sum test was employed for data that were ordinal or not normally distributed. Pearson's correlations were used to test for linear relationships between variables measured on an interval scale. SAS, version 9.2, was used for all analyses. A two-tailed *P* value of less than 0.05 was considered statistically significant.

## 3. Results

Seventeen surgeons participated in the study and performed a simulated LVHR on an *in vivo* porcine model using Ventralight ST Mesh with transfascial sutures and Ventralight ST Mesh with Echo PS. The average number of years in practice for participating surgeons was 12.2 (Table 1). The surgeon population reported a varied practice background including community hospitals, academic centers, solo and group practice, and hospital employee. Just under half (8/17 surgeons: 47%) of the surgeon population reported advanced fellowship training in laparoscopy or minimally invasive surgery. All surgeons reported hernia repair as a significant portion of their practice and on average performed 40 ventral hernia repairs per year. Surgeons reported that an average of 86% of their LVHR had been performed laparoscopically in the past 12 months. 

**Figure 7:**
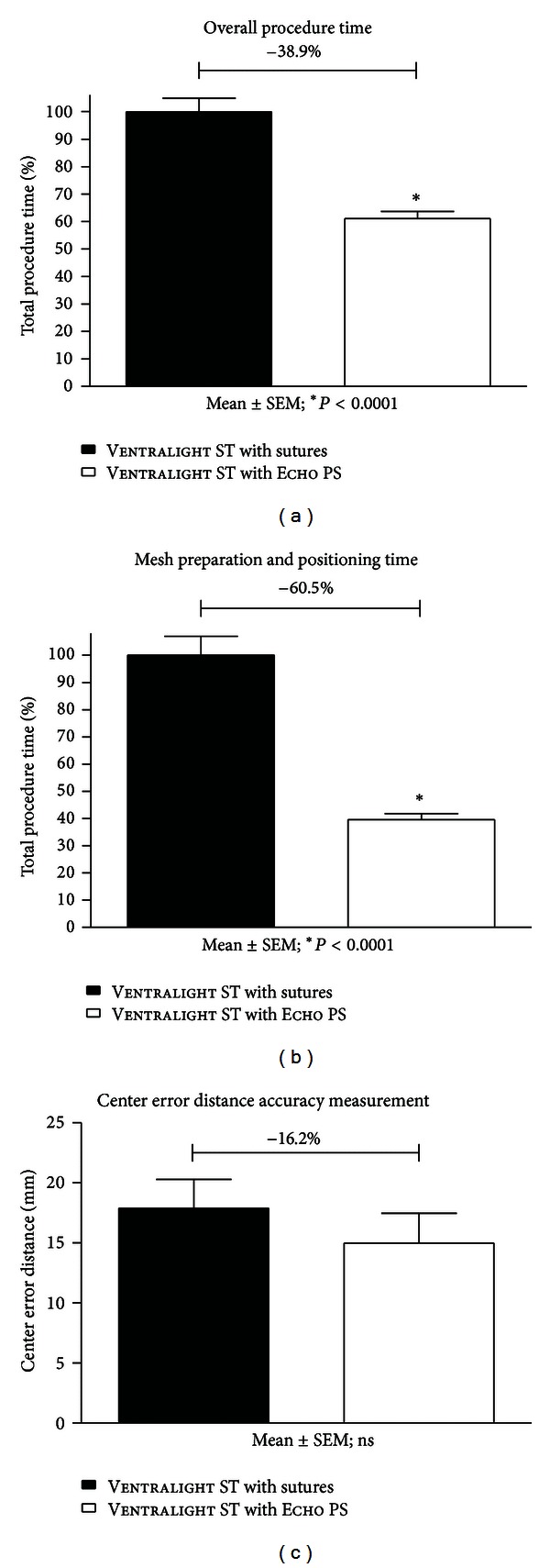
(a) Percentage of overall mesh procedure time, (b) mesh preparation and positioning time, and (c) center error distance accuracy Measurement. Ventralight ST Mesh with Echo PS demonstrated a significant 38.9% reduction in overall mesh procedure time, of which a 60.5% reduction in time was specifically associated with the mesh preparation and positioning phase of the procedure. Furthermore, a trend towards an improvement in centering accuracy was observed for Ventralight ST Mesh with Echo PS (16.2% improved centering accuracy); however, this was not statistically significant. Mean ± SEM; *P < 0.0001.

Overall time savings with use of Echo PS in simulated LVHR were 38.9% of total time, as compared to the transfascial suture technique employed (Figure 7(a)). The mesh positioning portion of the procedure was associated with time savings of 60.5% (Figure 7(b)). Accuracy in placement of the mesh over the center of the defect was recorded as the center error distance by the measurement of the vector from the center of the simulated surgical defect to the center of the placed mesh. With the best possible accuracy score (center error distance score) of zero, mesh centering accuracy was improved by 16.2% with the use of Echo PS (Figure 7(c)), although the results were not statistically significant.

Procedure time variability was analyzed by evaluating the standard error (SE) of the mean. The variability was greater for Ventralight ST Mesh with transfascial sutures (SEM: 4.9%), as compared to Ventralight ST Mesh with Echo PS (SEM: 2.7%), resulting in 84.2% less variability for Ventralight ST Mesh with Echo PS, as compared to Ventralight ST Mesh with transfascial sutures.

## 4. Discussion

 The operating room is one of the highest cost of care settings in the hospital, which represents a challenge yet also an opportunity for improvement and efficiency. Multiple studies have previously demonstrated efficiency improvement in the operating room by correcting patient work flow processes including streamlining preoperative checks and anesthesia consultation, improving faster turnover times, and developing procedure specific teams with assigned and defined roles and tasks leading to improved efficiency [7, 8]. One alternative area for efficiency improvement in the operating room may be to focus on surgical techniques that save time or reduce procedure variability. Historically, surgeons are trained for movement efficiency in order to perform procedures quickly and safely. It comes as no surprise that new technology may help reduce procedure time and improve overall operative efficiency. 

In this study, we evaluated the use of Echo PS mesh in simulated LVHR, as compared to a standard repair technique using four corner transfascial sutures for mesh positioning and initial fixation. We found a 39% reduction in overall time to complete LVHR in the porcine model. Of the overall time savings, a 61% reduction in operative time was demonstrated during the preparation, insertion, and positioning phase of the procedure. The significant time savings demonstrated for the Echo PS group, particularly during one of the most challenging (positioning) phases of the procedure in this preclinical model, provide validation for the utility of a detachable balloon positioning device in LVHR. A trend towards centering accuracy was also observed for Echo PS; however, this was not statistically significant. 

We believe that the significant time savings observed within this preclinical study may have the potential to translate into an overall operative cost savings for LVHR. Previous studies have evaluated direct cost savings due to decreased time spent in the OR based upon an estimate of cost per minute in the operating room. From multiple reports, associated cost per minute in the operating room ranges from $15 to 66 per minute [9–13]. However, the range of costs in this estimate may largely be due to the complexity of underlying patient conditions and the level of anesthesia monitoring in these series. In addition, based upon the complexity of the surgical center and individual hospital associated costs, the cost per minute in operating room varies drastically from location to location. Perhaps a more accurate estimation of total value would be to use the opportunity cost per minute as defined as the value of an activity foregone for participation in another activity [14, 15]. In a cost analysis of laparoscopic versus open colectomy, Chatterjee and colleagues used opportunity cost to demonstrate that the average time of 27 additional minutes spent performing a laparoscopic colectomy as compared to open colectomy equates to a missed opportunity cost of $250–700 [16]. With an average procedure time savings of almost 40% with the use of Echo PS, the equated monetary value of operative time savings may range from hundreds to thousands of dollars (based upon the average operating room costs per facility). However, the opportunity cost for the surgeon may equate to a much higher value as the relative decrease in time may allow the surgeon to schedule additional procedures or more complex procedures with higher relative value units. 

Aside from the monetary aspect, reduced operative procedure time is associated with other important benefits. Primarily, we acknowledge the potential to reduce general anesthetic time for the patient which, particularly for those patients with multiple medical comorbidities, could be translated to an improvement in operative safety. The literature suggests that shorter anesthesia durations may be associated with reduced postoperative infection rates [17], reduced postoperative nausea and vomiting [18], reduced pulmonary complications [19], and reduced length of stay [17].

Echo PS was also found to decrease procedure time variability by 84%, indicating that procedure time with Echo PS was more consistent across surgeons than doing the procedure with transfascial positioning sutures. We speculate that more consistent procedure times may lead to greater operating efficiencies during patient procedures, reduced start time tardiness, fewer excess staffing costs, and potentially even better results on patient satisfaction surveys [20]. The decrease in time spent to perform LVHR in addition to reduced procedure time variability may improve the overall workflow efficiency and allow for more laparoscopic procedures to be performed in the same time period. 

While the study design was intended to realistically simulate LVHR, we recognize the unique degree of efficiency within every operating room environment. We have attempted to control this by using identical operative setups with the same operative staff in the same surgical suite. We also acknowledge that some intrasurgeon variability may exist and have attempted to negate this variability by having each surgeon serveing as their own control (i.e. by performing both techniques). 

## 5. Conclusions

The use of Echo PS for simulated LVHR in a porcine model resulted in a statistically significant reduction in overall procedure time and a decrease in procedure time variability. In particular, a considerable amount of time savings was demonstrated during intracorporeal mesh placement and orientation within the abdomen. We believe that Echo PS will continue to demonstrate improved efficiency in clinical practice. Findings from this preclinical study helps to support the improved LVHR procedural efficiency which may translate into shorter general anesthesia time for patients and streamlined healthcare delivery. The time savings realized by use of this device may demonstrate direct cost savings through reduced operating room-associated and procedure-related costs or indirectly through improved opportunity costs for surgeons. Ultimately, additional clinical followup is necessary to demonstrate long-term efficacy and equivalently low rates of hernia recurrence with prospective analysis of procedure related cost comparison between traditional mesh positioning techniques and the use of Echo PS for LVHR.

## Figures and Tables

**Table 1:** Participating surgeon demographics.

	*N* = 17
Age (years), mean	45.9
Years in practice, mean (range)	12.2 (2–37)
Years of laparoscopy practice, mean (range)	11.4 (2–22)
Completed advanced laparoscopic or minimally invasive training, *N*	8
Number of hernia repairs done in the previous 12 months (any type), mean (range)	142.6 (25–400)
Ventral hernia repairs, mean (range)	40 (20–115)
Percentage of ventral hernia repairs performed laparoscopically, mean (range)	86% (35–100)
